# Innovative, Technology-Driven, Digital Tools for Managing Pediatric Urinary Incontinence: Scoping Review

**DOI:** 10.2196/66336

**Published:** 2025-05-05

**Authors:** Lola Bladt, Jiri Vermeulen, Alexandra Vermandel, Gunter De Win, Lukas Van Campenhout

**Affiliations:** 1 Department of Research and Development Minze Health NV Antwerp Belgium; 2 Department of Product Development Faculty of Design Sciences University of Antwerp Antwerp Belgium; 3 Department of Rehabilitation Sciences and Physiotherapy Faculty of Medicine and Health Sciences University of Antwerp Antwerp Belgium; 4 Department of Urology University Hospital Antwerp Edegem Belgium; 5 Antwerp Surgical Training, Anatomy and Research Centre (ASTARC) Faculty of Medicine and Health Sciences University of Antwerp Antwerp Belgium

**Keywords:** pediatric urinary incontinence, nocturnal enuresis, behavioral therapy, urotherapy, patient compliance, digital health, serious games, telehealth, health technology, enuresis alarm, artificial intelligence, AI

## Abstract

**Background:**

Urinary incontinence affects approximately 7% to 10% of children during the day and 9% to 12% of children during the night. Treatment mainly involves lifestyle advice and behavioral methods, but motivation and adherence are low. Traditional tools such as pen-and-paper solutions may feel outdated and no longer meet the needs of today’s “digital native” children. Meanwhile, digital interventions have already shown effectiveness in other pediatric health care areas.

**Objective:**

This scoping review aimed to identify and map innovative, technology-driven, digital tools for managing pediatric urinary incontinence.

**Methods:**

PubMed, Web of Science, and the Cochrane Library were searched in March 2022 without date restrictions, complemented by cross-referencing. Studies were eligible if they focused on pediatric patients (aged ≤18 years) with bladder and bowel dysfunctions and explored noninvasive, technology-based interventions such as digital health, remote monitoring, and gamification. Studies on adults, invasive treatments, and conventional methods without tangible tools were excluded. Gray literature was considered, but non–English-language, inaccessible, or result-lacking articles were excluded. A formal critical appraisal was not conducted as the focus was on mapping existing tools rather than evaluating effectiveness. Data analysis combined descriptive statistics and qualitative content analysis, categorizing tools through iterative coding and team discussions.

**Results:**

In total, 66 articles were included, with nearly one-third (21/66, 32%) focusing on nocturnal enuresis. Our analysis led to the identification of six main categories of tools: (1) digital self-management (7/66, 11%); (2) serious games (7/66, 11%); (3) reminder technology (6/66, 9%); (4) educational media (12/66, 18%), further divided into video (5/12, 42%) and other media (7/12, 58%); (5) telehealth and remote patient monitoring (13/66, 20%), with subcategories of communication (5/13, 38%) and technological advances (8/13, 62%); and (6) enuresis alarm innovations (21/66, 32%), further divided into novel configurations (8/21, 38%) and prevoid alarms (13/21, 62%).

**Conclusions:**

The field of pediatric urinary incontinence demonstrates a considerable level of innovation, as evidenced by the inclusion of 66 studies. Many tools identified in this review were described as promising and feasible alternatives to traditional methods. These tools were reported to enhance engagement, improve compliance, and increase patient satisfaction and preference while also having the potential to save time for health care providers. However, this review also identified gaps in research, highlighting the need for more rigorous research to better assess the tools’ effectiveness and address the complex, multifaceted challenges of pediatric urinary incontinence management. Limitations of this review include restricting the search to 3 databases, excluding non–English-language articles, the broad scope, and single-reviewer screening, although frequent team discussions ensured rigor. We propose that future tools should integrate connected, adaptive, and personalized approaches that align with stakeholder needs, guided by a multidisciplinary, human-centered framework combining both qualitative and quantitative insights.

## Introduction

### Background

Urinary incontinence (UI) is prevalent among children, affecting approximately 7% to 10% (aged 5-13 years) during the day [1] and 9% to 12% (aged 5-15 years) during the night [2-4]. UI often leads to low self-esteem, shame, and stress, negatively impacting the child’s psychosocial well-being and overall quality of life [5-9]. The primary treatment approach for pediatric UI, once anatomical and neurogenic causes are excluded, mainly involves lifestyle advice and behavioral methods [10-12]. These methods vary depending on whether the UI occurs during the day or at night.

For daytime UI (DUI), the recommended first-line approach is standard urotherapy. It encompasses patient education, keeping track of symptoms through bladder diaries, regulating fluid intake and toilet routines, adhering to scheduled voiding, and providing support and encouragement [13-16]. An important part of this treatment is teaching children appropriate responses to their bladder signals. Standard urotherapy achieves success rates ranging from 40% to 56% [17-19]. For nighttime UI, also known as nocturnal enuresis (NE), one of the recommended first-line treatments is NE alarm training [20]. This method uses a moisture sensor that triggers an alarm to wake the child upon bed-wetting. Through this approach, children are conditioned to recognize and respond to bladder signals, ultimately helping reduce bed-wetting episodes. The success rate of alarm training is between 50% and 70% [21]. However, motivation and adherence, both crucial for these behavioral methods to be successful [22,23], are low in practice [22,24,25].

There are many factors that influence motivation and adherence [15,24-27], including duration of therapy, required effort, symptom bother, and awareness but also the supporting tools’ usability and acceptance. The evaluation and management of pediatric UI traditionally rely on pen-and-paper bladder diaries [28-30], along with verbal and written instructions for behavioral techniques [16] and traditional NE alarms [31]. However, these analogue methods may seem outdated to today’s “digital native” children, who are accustomed to using technology and digital tools [32]. Consequently, traditional approaches to pediatric UI management may no longer meet today’s children’s needs and expectations, potentially affecting motivation and adherence. Meanwhile, digital interventions have already proven effective as alternatives to traditional methods in other pediatric health care areas such as pain tracking [33], attention-deficit/ hyperactivity disorder therapy [34], childhood obesity prevention [35], and asthma management [36].

### Objectives

Therefore, our objective was to identify and map innovative, technology-driven, digital tools for pediatric UI management as alternatives to conventional approaches such as pen-and-paper solutions. In addition, we aimed to provide an overview of these tools’ characteristics, including their features, design drivers, and any reported evidence supporting their use.

## Methods

This scoping review followed the PRISMA-ScR (Preferred Reporting Items for Systematic Reviews and Meta-Analyses extension for Scoping Reviews) guidelines as outlined by Tricco et al [37] (see Multimedia Appendix 1 for the PRISMA-ScR checklist).

### Eligibility Criteria

Eligibility criteria were determined based on the population, concept, and context framework. The *population* for this review included pediatric patients experiencing conditions such as bladder and bowel dysfunction (BBD), NE, overactive bladder (OAB), underactive bladder, and dysfunctional voiding (DV), as well as neurogenic conditions such as spina bifida. Studies involving adult populations (aged ≥18 years) or unrelated conditions (eg, cancer or rare diseases) were excluded.

The *concept* focused on innovative, technology-driven, digital tools for managing pediatric UI and related dysfunctions. Eligible tools included smartphone apps, multimedia resources, and remote monitoring technologies, whereas conventional methods or treatment plans without tangible tools were excluded.

The *context* was limited to studies that addressed noninvasive therapeutic approaches, particularly behavioral methods such as urotherapy, bladder training, and alarm training. Invasive approaches, including surgeries and their associated tools, were excluded.

All types of literature were considered, including gray literature (eg, conference proceedings, preprints, and technical reports), to provide a broad range of available literature and reduce publication bias. However, articles not presenting results or findings, not written in English, or lacking full-text availability were excluded.

### Search Strategy

The search (see Multimedia Appendix 2 for the full search strategy) was conducted in March 2022 across 3 databases—PubMed, Web of Science, and the Cochrane Library—with no limitations on publication dates. The population, concept, and context framework guided the selection of search terms.

Key search terms for *population* included “children,” “adolescents,” “pediatric,” “bladder bowel dysfunction,” and “enuresis.” For *concept*, terms such as “digital,” “mHealth,” “gamification,” “multimedia,” and “remote monitoring” were used. *Context*-related search terms included “urotherapy,” “bladder training,” “alarm therapy,” “self-monitoring,” and “behavior management.”

### Study Selection

The study selection process was conducted by the first author (LB), with consultation and supervision provided by the team of authors. After removing duplicates, potential studies underwent screening based on their titles and abstracts in line with the eligibility criteria, with any uncertainties discussed with the author team.

Subsequently, LB read and assessed the full-text articles, periodically consulting with the team to ensure consistency and rigor. To reduce selection bias from database choice, we conducted cross-referencing, scanning the reference lists of the included articles to identify additional relevant studies.

The selected studies underwent final review and confirmation by the entire author team. A formal critical appraisal or methodological quality assessment was not conducted as the aim of this scoping review was to identify and map the available evidence on innovative, technology-driven, digital tools rather than draw formal conclusions on their effectiveness.

### Data Extraction

Data were extracted and charted using Microsoft Excel (Microsoft Corp) according to four main heading groups: (1) article information, (2) participant characteristics (population), (3) tool description (concept), and (4) research methods and results (context). Article information encompassed details such as article title, authors, journal, publication year, country, and database source. Participant characteristics included sample size, age range, sex distribution, and dysfunction indication. Tool description covered intended therapy type, tool design details, and its design drivers. Finally, research methods and results comprised study design, intervention groups, study outcomes, and conclusions.

### Data Analysis

A combination of descriptive statistics and basic qualitative content analysis was used for data analysis. Descriptive statistics were used to analyze study characteristics, providing frequency counts and proportions for extraction items such as study design and dysfunction indication. Basic qualitative content analysis was used to identify and classify innovative, technology-driven, digital tools into distinct categories. The process began with multiple readings of the extraction items related to the tool description to familiarize ourselves with the data. Following an inductive approach, initial notes were made to generate codes, and coding was manually conducted in Microsoft Excel, where relevant terms were highlighted and grouped into emerging patterns. Through iterative refinement and team discussions, codes were merged, removed, or redefined to eliminate overlap and ensure sufficient distinction. The final set of codes was organized into 6 broad categories, 3 of which were further subdivided to capture more specific aspects. LB conducted the preliminary analysis, and to ensure consistency and reliability, the coding process was reviewed and refined collaboratively by the coauthors at multiple stages. Any disagreements on categorization were addressed through discussions to achieve consensus.

## Results

### Retrieval of Studies

**Figure 1:**
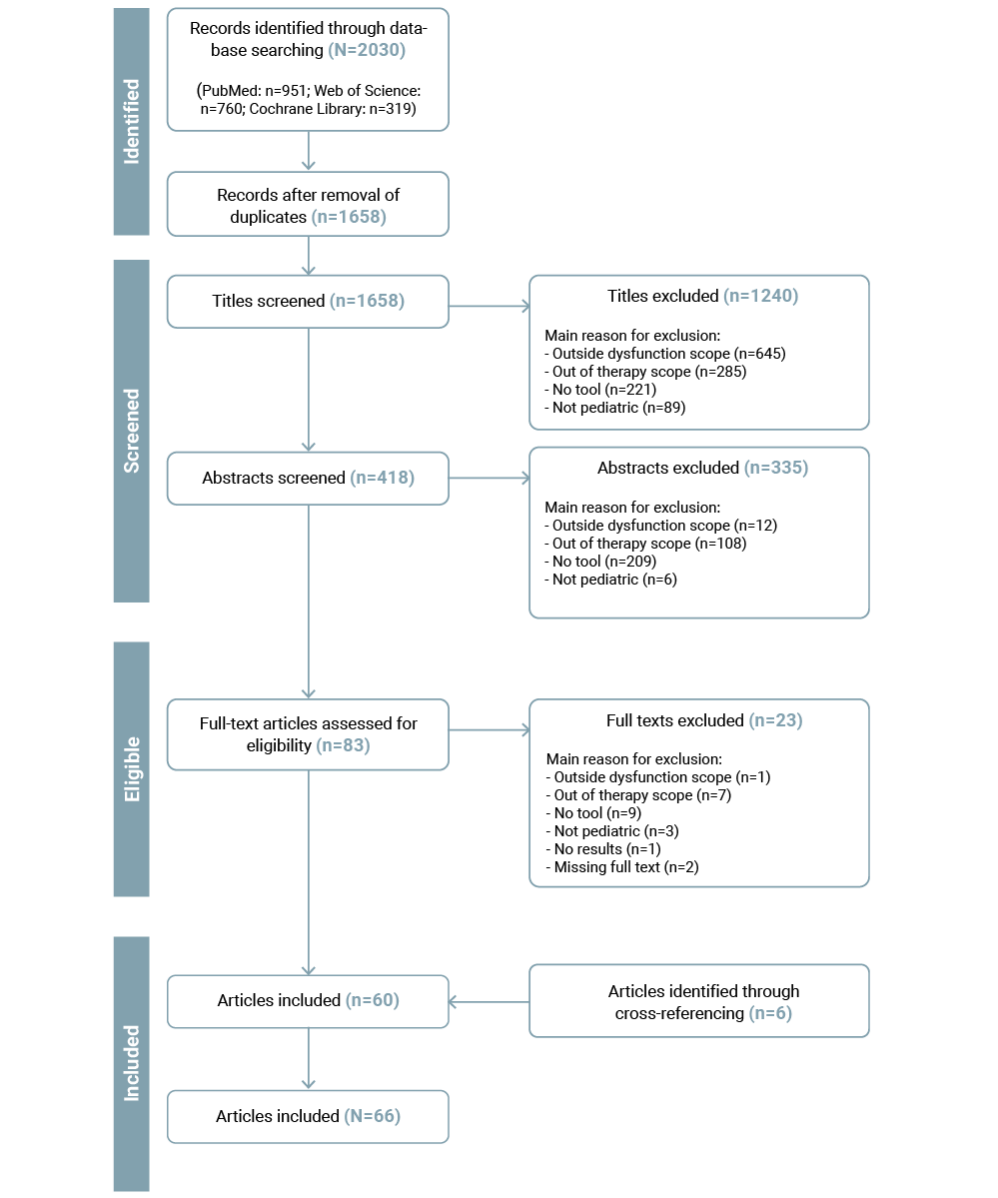
PRISMA (Preferred Reporting Items for Systematic Reviews and Meta-Analyses) flowchart of the literature screening and selection process.

The search retrieved a total of 2030 articles (PubMed: n=951, 46.85%; Web of Science: n=760, 37.44%; Cochrane Library: n=319, 15.71%). After removing duplicates (372/2030, 18.33%), a total of 81.67% (1658/2030) of the studies were included in the title and abstract screening. Following title and abstract screening, of the 1658 articles, 1575 (94.99%) were excluded, leaving 83 (5.01%) for full-text evaluation. Finally, 60 articles were included in the data extraction process, with an additional 6 identified through cross-referencing, resulting in a total of 66 included articles. Figure 1 illustrates the flowchart of the screening and selection process.

### General Analysis to Describe the Study Characteristics

Of the included studies (n=66), most were randomized controlled trials (RCTs; 14/66, 21%) and validation studies (13/66, 20%), followed by quasi-experiments (10/66, 15%), prospective studies with no control group (7/66, 11%), and qualitative or mixed methods studies (8/66, 12%), as shown in Table 1. Nearly one-third (21/66, 32%) of the identified tools targeted children with NE, followed by DV (9/66, 14%), BBD (7/66, 11%), and DUI (7/66, 11%). More than one-third (25/66, 38%) of the studies evaluated outcomes related to patient experience, such as satisfaction, motivation, and usability. A mean sample size of 82 (SD 83; range 14-353) participants was found for the RCTs, quasi-experiments, and prospective studies (no control), indicating a large variation in sample size.

**Figure 2:**
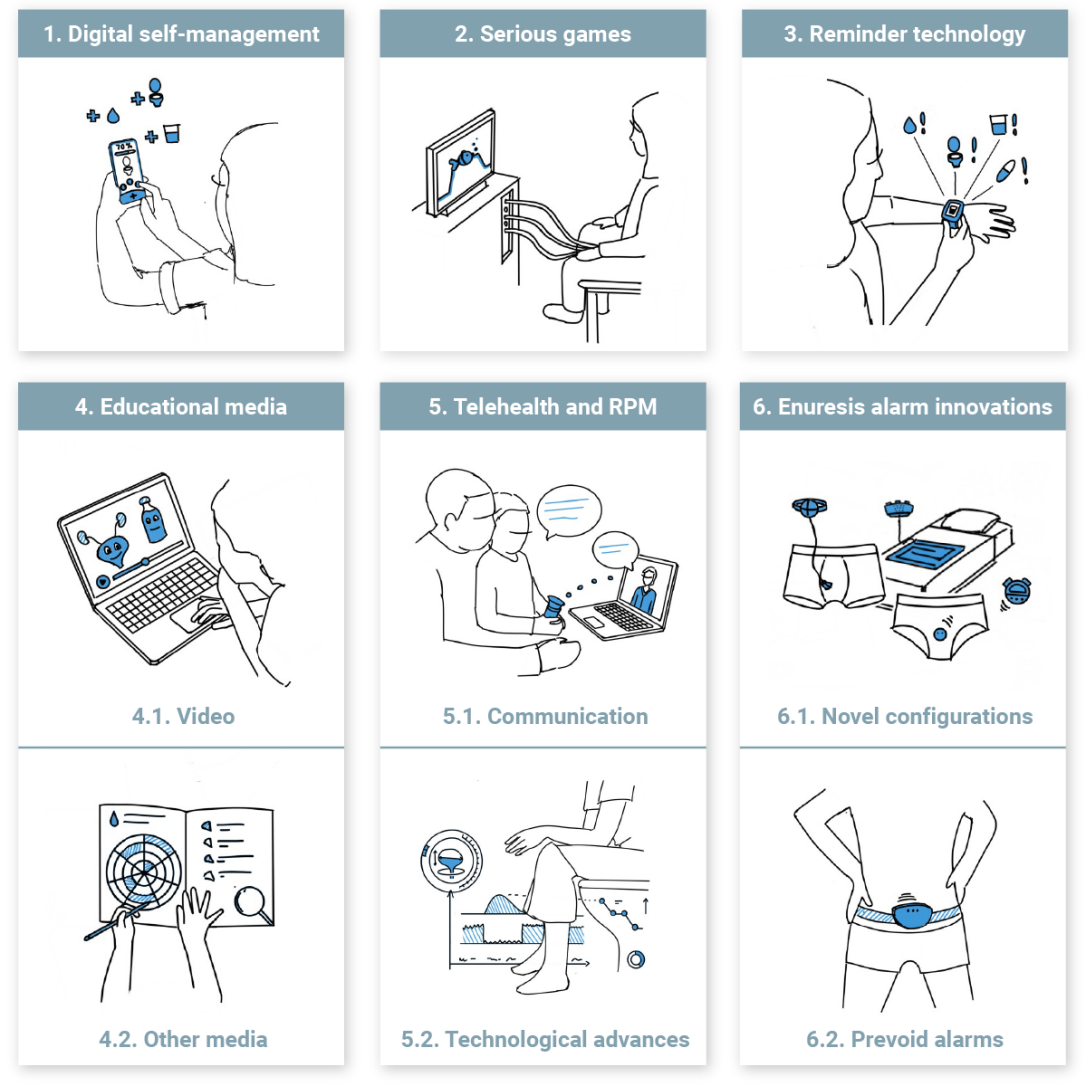
Overview of the 6 categories and subcategories of innovative, technology-driven, digital tools. RPM: remote patient monitoring.

We identified and established a total of six primary categories of innovative, technology-driven, digital tools (Figure 2): (1) digital self-management (7/66, 11%), (2) serious games (7/66, 11%), (3) reminder technology (6/66, 9%), (4) educational media (12/66, 18%), (5) telehealth and remote patient monitoring (RPM; 13/66, 20%), and (6) enuresis alarm innovations (21/66, 32%). Further subcategories were identified within educational media, including video (5/12, 42%) and other media (7/12, 58%); within telehealth and RPM, including communication (5/13, 38%) and technological advances (8/13, 62%); and within enuresis alarm innovations, including novel configurations (8/21, 38%) and prevoid alarms (13/21, 62%).

**Table 1:** Summary of study characteristics (n=66).

Study characteristic		Studies, n (%)
**Study design**		
	RCT^a^	14 (21)
	Feasibility or validation study	13 (20)
	Quasi-experiment	10 (15)
	Prospective nc^b^	7 (11)
	Review	6 (9)
	Mixed methods study	5 (8)
	Retrospective study	4 (6)
	Qualitative study	3 (5)
	Modeling	2 (3)
	Case study	1 (2)
	Design-based research	1 (2)
**Dysfunction indication**		
	NE^c^	21 (32)
	DV^d^	9 (14)
	BBD^e^	7 (11)
	DUI^f^	7 (11)
	DUI or nighttime UI^g^	5 (8)
	Neurogenic	5 (8)
	UTI^h^	4 (6)
	Developmental or neurodevelopmental disorder	3 (5)
	OAB^i^	2 (3)
	UAB^j^	1 (2)
	Bowel dysfunction	1 (2)
	Anatomical	1 (2)

^a^RCT: randomized controlled trial.

^b^nc: no control.

^c^NE: nocturnal enuresis.

^d^DV: dysfunctional voiding.

^e^BBD: bladder and bowel dysfunction.

^f^DUI: daytime urinary incontinence.

^g^UI: urinary incontinence.

^h^UTI: urinary tract infection.

^i^OAB: overactive bladder.

^j^UAB: underactive bladder.

Overall, we found that the distribution of dysfunction types was generally well balanced across the different tool categories. Digital self-management exhibited a diverse distribution, addressing a range of dysfunctions including BBD (1/7, 14%), DUI or nighttime UI (1/7, 14%), and neurogenic disorders (2/7, 29%). Serious games were predominantly used for DV (5/7, 71%). Reminder technology was used for timed voiding in cases of DUI and OAB (3/6, 50%), as well as for self-management of neurogenic and developmental or neurodevelopmental disorders (3/6, 50%). Educational media was used across a range of dysfunctions, with slightly more use for NE (4/12, 33%) and urinary tract infections (UTIs; 3/12, 25%). Telehealth was primarily used for BBD (4/13, 31%) and DUI or nighttime UI (4/13, 31%), with additional use also observed for DV (3/13, 23%). Finally, enuresis alarm innovations were predominantly associated with NE (16/21, 76%), as expected, although not exclusively. They were also applied for other conditions, such as DUI (2/21, 10%) and developmental or neurodevelopmental disorders (2/21, 10%).

### Categories of Innovative, Technology-Driven, Digital Tools

#### Digital Self-Management

##### Category Overview and Key Drivers

Digital devices such as smartphones, tablets, and computers have become increasingly prevalent in our society, especially among children and adolescents [38]. Therefore, they offer a practical and accessible medium with a range of health care possibilities, such as digital self-management to enhance patient engagement and improving access, availability, and continuity of care [38,39].

##### Tools Within This Category

This review identified 11% (7/66) of the studies on digital self-management, with extended details available in Table S1 in Multimedia Appendix 3 [40-105]. These included electronic bladder diary apps [40,41], which served as digital alternatives to traditional paper versions to improve patient compliance. In addition, 3 self-management applications with training programs were included, targeting conditions such as DUI (smartphone app) [42], bowel dysfunctions (internet application) [43], and spina bifida (smartphone app) [44,45]. These self-management applications incorporated features such as weekly modules with exercises; daily goals; self-monitoring of parameters such as fluid intake, toilet visits, medication, and mood status; reminders; progress tracking; personalized feedback; and rewards such as in-application trophies and stars. Furthermore, a parent software portal [46] was identified, enabling parents to monitor their children’s symptoms, such as UTIs, to aid clinical decision-making.

##### Study Results and Conclusions

The studies on digital self-management tools reported feasibility, acceptability, ease of use, and patient and parent satisfaction [40-46]. Furthermore, they described improvements in patient compliance, as observed in both the bowel dysfunction self-management program and the parent software portal, with high completion rates and low dropout [43,46]. However, the parent software portal was described as facing challenges in effectively delivering information to health care professionals, lacking integration into their busy clinical practice [46].

Despite many promising aspects, the studies often reported limitations in providing robust evidence of effectiveness. For example, the electronic bladder diary format was described as offering data quality advantages for certain patients but was not found to be superior to a paper-based bladder diary [40]. The sole RCT investigating a self-management program for bowel dysfunctions reported greater improvements in patients’ quality of life and reduction in overall symptom severity. However, this was in comparison to a nonequivalent control group of children on a waitlist who were not receiving any treatment [43].

#### Serious Games

##### Category Overview and Key Drivers

Serious games are interactive applications designed as games but intended for educational or therapeutic purposes. By blending elements of fun and seriousness, serious games offer a playful way for children to learn about complex topics such as bladder and bowel health. This approach can be particularly effective for engaging children, who have limited attention spans and may struggle with processing information. Moreover, the entertainment aspect of serious games is crucial for motivating and engaging children as they strive for success within the game [41,106,107].

##### Tools Within This Category

Table S2 in Multimedia Appendix 3 [40-105] shows that serious games were mainly used for pelvic floor biofeedback training through biogames (6/7, 86%) [47-52]. In these biogames, the game action was controlled by the appropriate muscular contractions and relaxations detected by pelvic floor electromyography (EMG) patches worn by the child. Various animated scenarios were available, such as golfing, flying spaceships, riding an elephant, playing basketball, and swimming with dolphins or fish. In addition, one serious game was found for standard urotherapy, which was played on a smartphone [53]. In this game, the child was rewarded for maintaining a daily bladder diary and voiding adequately during the 3-month training program, with additional rewards for staying dry.

##### Study Results and Conclusions

Overall, the studies described positive effects of biogames for pelvic floor biofeedback training on health outcomes, particularly for children with DV [47-50,52] and, in one study, for those with underactive bladder [51]. However, understanding the precise impact of adding a game to the therapy is challenging as the studies reported limitations such as retrospective designs [49], lack of control groups [47], and nonequivalent therapy comparisons [48,50,51].

Findings varied across the studies. One study reported similar results in half the time with biogames (mean of 3.6, SD 1.8 sessions) compared to without (mean of 7.6, SD 5.2 sessions) [49], whereas another study reported a relatively high number of treatment sessions with biogames (mean of 9.6, SD 1.3 sessions) [50]. In the sole RCT comparing equivalent therapies [52], no differences were observed in terms of clinical success, cooperation, and motivation between pelvic floor biofeedback training with and without biogames. Nevertheless, subjective observations suggested that using biogames effectively captured children’s attention and prevented boredom during the sessions, particularly among younger children [52].

Similarly, in the comparison of standard urotherapy with and without a serious game, no significant differences were reported in training results, quality of life, or motivation [53]. However, children expressed a clear preference for the game. They provided valuable suggestions for its improvement, such as introducing more dynamic gameplay with changing storylines and evolving game types, along with features such as regular updates on training progress and detailed information about their bladder problem.

#### Reminder Technology

##### Category Overview and Key Drivers

Reminder technology uses programmable timers to activate alarms aimed at assisting children in adhering to therapy or self-management schedules without requiring constant prompting from parents. This approach supports children’s independence as parental reminders may sometimes contribute to resistance, with children preferring autonomy in adhering to their schedules [108].

##### Tools Within This Category

A total of 9% (6/66) of the studies were categorized under reminder technology (Table S3 in Multimedia Appendix 3 [40-105]). Half (3/6, 50%) of these studies evaluated timer watches, which were used in standard urotherapy to assist with timed voiding [54-56]. The timer watch can be programmed to automatically alarm at specified intervals, such as every 2 hours, helping children maintain a consistent voiding schedule.

The other half (3/6, 50%) of the studies focused on self-management reminders for children with neurogenic or developmental or neurodevelopmental disorders [57-59]. A laptop calendar program reminded children with prospective memory disabilities to complete and log daily schedules, such as self-catheterization and medication, through visual and auditory modalities [59]. A smartwatch with a built-in fitness tracker displayed visual icons to prompt children with spina bifida to engage in various daily activities from self-care to playtime and rewarding timely completion with stars [58]. Finally, “Potty Monkey,” an interactive toy, prompted children with special needs to void at set times, providing reminders, modeling techniques, and positive reinforcement for time-based toilet training [57].

##### Study Results and Conclusions

Reminder technology for timed voiding using a timer watch was reported to be superior in treating severe DUI in children with OAB compared to standard urotherapy without it, with improved adherence to the voiding schedule playing a key factor [55,56]. In addition, another study described reminder technology as an equally effective but simpler alternative to enuresis alarms (using moisture sensors) in treating DUI, also reporting good adherence rates [54].

For self-management in children with neurogenic or developmental or neurodevelopmental disorders, reminder technology was reported to be feasible, although the findings were based on small sample sizes [57-59]. The tools were described as alleviating anxiety in caregivers and patients by providing a log of tasks [59] and fostering child independence through clear routines [58]. However, patient acceptance varied, with the smartwatch being highly accepted [58] and the interactive toy receiving lower acceptance [57], with aesthetics and flexibility playing a key role.

#### Educational Media

##### Video

###### Category Overview and Key Drivers

Videos, as educational media, offer a visually engaging and interactive format for explaining complex concepts and techniques to patients and parents. They provide a convenient and 24/7-available alternative or supplement to in-person visits. Moreover, videos offer anonymity, which can be beneficial for individuals who feel embarrassed or hesitant to seek medical advice in person. As a result, the internet and video-sharing platforms have become popular sources of health-related information [32].

###### Tools Within This Category

In our review, 8% (5/66) of the studies were classified under the subcategory of video within educational media (Table S4 in Multimedia Appendix 3 [40-105]). These videos covered a range of topics. Some supported behavioral therapy, such as an animated bladder training video [60,61] and an abundance of YouTube videos on NE therapy [62,63]. Another video focused on preparing children for surgical interventions, featuring actual hospital staff and using role-play modeling techniques to demonstrate the anesthesia process [64].

###### Study Results and Conclusions

Multiple studies within this subcategory (3/5, 60%) described videos as effective educational media for patients offering alternatives to in-person strategies [60,61,64]. In a noninferiority RCT, the animated bladder training video was found to be equivalent to standard urotherapy in reducing bladder and bowel symptoms [60] and improving quality of life scores [61]. Similarly, the presurgery video was reported to effectively reduce preoperative anxiety and resulted in less postoperative maladaptive behavior [64].

While videos were described as effective tools for educating patients and families, caution is recommended when relying on video-sharing platforms such as YouTube that lack robust systems to ensure the accuracy, completeness, and quality of the information [62,63]. The studies indicated a need for a balance between adherence to evidence-based guidelines and the creation of engaging content that captures and maintains viewer attention.

##### Other Media

###### Category Overview and Key Drivers

Effective information delivery is crucial for raising awareness, setting expectations, promoting patient adherence, and ensuring data quality. Unfortunately, verbal explanations by physicians may be poorly understood due to time constraints and complexity, whereas written information may not always be suitable or desirable for children, resulting in limited child participation. Thus, using other types of educational media tailored and engaging for children may help ensure effective information delivery to pediatric patients.

###### Tools Within This Category

A total of 11% (7/66) of the studies were classified under other types of educational media (Table S5 in Multimedia Appendix 3 [40-105]). This category included 2 homework books for UI: one that used coloring activities to log the severity of incontinence [65] and another that featured practical exercises for standard urotherapy [66]. In addition, a multimedia program for NE was identified, which combined text, drawings, cartoons, and sound to educate children on bed-wetting [67,68]. Finally, educational materials on UTIs included educational urine kits for clean-catch urine collection [69-71] and a low-cost origami-based microscope that used a mobile phone camera to raise awareness of genital hygiene and UTIs in schools [71].

###### Study Results and Conclusions

The UI homework books were reported to be feasible, usable, and well received, with good compliance [65,66]. In addition, the homework book for standard urotherapy was associated with increased fluid intake and hold time [66].

The multimedia program for NE was noted to show promise in holding children’s attention and improving their knowledge, especially for younger children with low literacy levels [67]. However, results from the RCT indicated that, while the multimedia program was not more effective than traditional verbal and written information, it could help save health care professionals time in patient education [68].

Similarly, urine kits with educational pamphlets for clean-catch urine collection did not result in a significant reduction in contamination rates but may help streamline patient education [69,70]. In addition, the origami-based microscope was described as a promising educational tool for promoting health awareness by detecting UTI-positive urine samples reasonably well [71].

#### Telehealth and RPM

##### Communication

###### Category Overview and Key Drivers

Telehealth and RPM enable remote communication and data exchange between patients and clinicians. Although face-to-face communication has been shown to improve treatment adherence [27,109,110], constraints such as time, cost, and travel can lead to extended intervals between consultations, potentially affecting patient motivation [111]. Telehealth may address some of these issues by offering consistent follow-up visits and motivational support without the need for physical visits.

###### Tools Within This Category

A total of 8% (5/66) of the studies were classified within the subcategory of communication using telehealth and RPM (Table S6 in Multimedia Appendix 3 [40-105]). One study explored videoconferencing for remote patient visits [72], whereas another study evaluated a UTI home monitoring system [73]. This system facilitated communication between parents of children with neurogenic bladder and urology nursing staff to alert them of potential UTI signs. It comprised a portable urinalysis device, Bluetooth weight scale, thermometer, electronic bladder diary, and mobile communication device. In addition, an embodied conversational agent, “Dr. Evie,” was identified, offering personalized lifestyle advice and empathetic coaching for UI self-management while generating reports for general practitioners [74-76].

###### Study Results and Conclusions

The studies reported high patient and parent satisfaction with telehealth, citing reduced stress and increased convenience, especially for rural families, due to decreased travel time [72-76]. In addition, the UI self-management program featuring an embodied conversational agent was reported to improve patient adherence rates from 50% to 76% on average and alleviate symptoms [74-76]. Advice from nonfamily members and a sense of alliance with Dr Evie were noted to enhance program engagement.

Health care providers expressed mixed feelings about telehealth, acknowledging improved access to care but reporting technical barriers in conducting physical examinations and establishing rapport, particularly with pediatric patients [72]. Integrating RPM systems such as the UTI monitoring system may mitigate these technical barriers by providing valuable data for remote support and enhancing patient and parent self-efficacy [73]. However, patients’ selective use of certain products, mainly the urinalysis device, was noted to create data gaps that hinder clinical evaluation. In addition, technical issues contributed to increased workload for clinical teams, emphasizing the importance of careful consideration of all stakeholders’ diverse needs and preferences to facilitate successful integration into clinical practice.

##### Technological Advances

###### Category Overview and Key Drivers

Technological advances enabling telehealth and RPM aim to overcome limitations of in-clinic procedures, including the psychological stress that children often experience in hospital settings [77,112]. These procedures can also be inconvenient and time-consuming, as seen with noninvasive uroflowmetry, which requires voiding with a full bladder [78]. Similarly, therapies such as EMG biofeedback demand significant time and financial resources due to poor compliance and the need for multiple visits [77]. Meanwhile, invasive studies carry health risks and are often discouraged, especially for young patients [113,114].

###### Tools Within This Category

In total, 12% (8/66) of the studies were classified in the telehealth and RPM subcategory that focused on technological advances (Table S7 in Multimedia Appendix 3 [40-105]). These ranged from a machine learning algorithm to predict enuresis in children [79] to a noninvasive alternative to traditional invasive urodynamics using near-infrared spectrometry [80].

Advances in uroflowmetry beyond traditional gravimetric methods for home use were also reviewed [78]. These included basic volumetric, mechanical, and electrical methods such as capacitance sensors. Unconventional approaches were also discussed, including image processing for drop patterns, ultrasound Doppler effect, vibration sensors on the penis, and acoustic technology using a smartphone app to record urine sound [78,81].

Several studies (5/8, 63%) explored adapting existing technologies for home use. This included gravimetric uroflowmetry for home use [77,81] and its application in therapy settings to provide biofeedback during voiding [77,82]. Another study introduced home pelvic floor biofeedback through a wearable EMG device featuring a watchlike design with a light scale to indicate muscle activity strength [83]. In addition, transcutaneous electrical nerve stimulation was evaluated as a user-friendly substitute for in-clinic percutaneous tibial nerve stimulation to enhance continence, enabling self-administration at home without invasive needle insertion [84].

###### Study Results and Conclusions

Overall, the studies described promising advances in technology. The machine learning technology was described as having promising accuracy in screening for enuresis, with older age at toilet training initiation identified as a significant predictor [79]. Both uroflowmetry and EMG biofeedback were described as effective in reducing symptoms in patients with DV [82,83], with home uroflowmetry biofeedback showing promise and patient preference [77]. Transcutaneous electrical nerve stimulation was reported to show efficacy in reducing NE episodes, providing a safe and noninvasive at-home treatment option [84].

However, some novel technologies such as near-infrared spectrometry were described as promising and well accepted by patients but were also noted to lack standardized benchmarks and face challenges in ensuring consistent and reliable data that align with established norms and guidelines [80]. Similarly, while various uroflowmetry methods exist, gravimetric uroflowmeters were favored due to their simplicity and reliability [78]. For example, sound-based uroflowmetry, despite showing strong correlations with conventional uroflowmetry, was reported to have wide limits of agreement, with its accuracy influenced by variables such as smartphone type and toilet settings [81].

#### Enuresis Alarm Innovations

##### Novel Configurations

###### Category Overview and Key Drivers

Enuresis alarms function by detecting urine on 2 conducting strips, causing a voltage change and triggering an alarm [85-87]. Alarms with rigid wires may cause discomfort or psychological effects for the child, whereas fragile sensors can lead to missed alarms due to unintentional or deliberate deactivation. Conversely, overly sensitive alarms may trigger false alarms, which can be discouraging for patients [86]. These design shortcomings related to comfort, durability, and reliability in traditional enuresis alarms are the reason for novel configurations.

###### Tools Within This Category

We encountered 12% (8/66) of the studies featuring various configurations of enuresis alarms (Table S8 in Multimedia Appendix 3 [40-105]), spanning pad-and-bell to body-worn alarms and wired to wireless alarms. The pad-and-bell alarm used a bed mat with conductive strips, whereas the body-worn alarm had a sensor attached to underwear [86-88]. Wireless versions transmitted signals to an alarm positioned further away, requiring the child to leave the bed to deactivate it [86]. Smart textile sensors integrated conductive threads into underwear or disposable urine pads [85], with recent innovations incorporating wireless transmitters and smartphone apps for notifications and reinforcement strategies [89].

Configurations of the alarm signal itself ranged from loud acoustic alarms to subtler options such as lights, vibrating alarms, or personalized recorded messages [86]. For instance, one study evaluated a code word alarm with a digital voice recorder for parents to record personalized code words, rewarding children for recalling them the following morning [90]. Finally, another, more unconventional approach involved electrical impulses via surface electrode pads to activate the external urethral sphincter to interrupt micturition instead of relying on audible alarms [91,92].

###### Study Results and Conclusions

Comparative studies on pad-and-bell versus body-worn alarms reported mixed results [87,88]. While one study reported better outcomes with the pad-and-bell alarm [88], another found both equally effective but noted high dropout rates due to embarrassment [87]. Variations in false alarm rates and missed alarms were observed, suggesting that individual device features may have influenced performance more than the alarm type itself.

A review [86] highlighted the potential of smart textiles to enhance comfort and detection efficiency, with studies reporting positive performance and safety during daytime training [85,89]. One of these studies specifically found that a smart textile alarm with an app delivered similar outcomes in less time with high patient and parent satisfaction compared to standard behavioral therapy for toilet training children with autism spectrum disorder [89].

Using a prerecorded code word was reported to improve arousal response and training attitude, reducing dropout by 10%, although it only affected treatment outcomes in a subgroup with monosymptomatic NE [90]. Electrical impulses were reported as effective with perineal placement [91], though based on a small sample, whereas suprapubic placement showed no significant effect [92]. Reported benefits included potentially reducing fully wet beds, boosting self-confidence, and family motivation, but drawbacks such as higher cost, complexity, and potential fear of electrical currents were also noted.

##### Prevoid Alarms

###### Category Overview and Key Drivers

Traditional enuresis alarms provide a warning after an enuretic incident has occurred. Consequently, both the child and the parent still experience the inconvenience and consequences of wet pants or sheets during treatment, requiring a substantial level of motivation [93,94] Moreover, the child must establish an indirect link between the full bladder sensation and the alarm as it sounds after the bladder has emptied. These limitations have driven the development of prevoid enuresis alarms, designed to anticipate enuretic incidents by predicting when they might occur and provide a warning in advance.

###### Tools Within This Category

A total of 20% (13/66) of the studies were found on prevoid alarms (Table S9 in Multimedia Appendix 3 [40-105]), of which most (12/13, 92%) used ultrasound technology to trigger an alarm when bladder volume reached a predefined threshold. Basic ultrasound technology detected the posterior bladder wall rising above the symphysis pubis [93,95], whereas advanced systems estimated bladder volume using an array of transducers to locate both the anterior and posterior bladder walls through ultrasound echoes [94,96-104].

Various specialized measures were implemented to enhance the reliability of prevoid alarms. This included using intelligent software and machine learning to customize for factors such as body morphology, age, and sex [95,98-104]. Furthermore, to mitigate sensitivity to ultrasound probe movement, solutions such as tailored belts [93,95,104], elastic garments [94,98-100], adhesive materials [101-103], and even synchronizing measurements with the child’s respiratory rhythm [95] were explored.

One prevoid alarm system estimated bladder volume using wearables and deep learning instead of ultrasound. It tracked impedance using a waist belt sensor, heart rate and sleep stage using a smartwatch, and limb movement using fabric foot bands [105].

###### Study Results and Conclusions

The studies on prevoid enuresis alarms were primarily validation and feasibility studies reporting on the technological feasibility of these alarms, with detection rates varying from <50% to 90% [93,95-98,102,104,105]. However, sample sizes were small (1 to 41 patients) or relied on artificial bladder models.

Patient acceptance, influenced by the need for a comfortable, discrete, and nonstigmatizing design, was evaluated for MyPad [99,100] and SENS-U [101,103], both of which were well accepted. SENS-U was also reported to be feasible for both daytime bladder training [101] and nighttime monitoring [103].

Despite feasibility, several challenges were noted, including sensor positioning sensitivity; bladder shape changes due to an enlarged rectum or posture; and compatibility issues with different body types, particularly in children with obesity [93,94,98,100-104]. In addition, daytime observations of SENS-U showed that 23% of children experienced urgency or leakage before reaching the predefined volume-based threshold [101].

Finally, only 8% (1/13) of these studies compared the prevoid alarm with the traditional alarm, reporting comparable cure rates but a treatment duration approximately 3 times longer with the prevoid alarm [94]. The study suggested that awakening to wet sheets may constitute a classic conditioning negative reinforcement potentially essential for the cure process.

## Discussion

### Principal Findings

In this scoping review, we identified and mapped innovative, technology-driven, digital tools for managing pediatric UI. The field of pediatric UI demonstrates a considerable level of innovation, as evidenced by the inclusion of 66 articles, with nearly one-third (21/66, 32%) focusing on NE. Our analysis led to the identification of six primary categories of tools, 3 of which were divided into subcategories. (1) Digital self-management tools, which leveraged the widespread use of smartphones, tablets, and computers, offering a convenient and accessible medium for health-related purposes such as electronic bladder diaries [40,41] and self-management apps or portals [42-46]. (2) Serious games, which engaged children through playful interactions, such as games for pelvic floor biofeedback training [47-52] and standard urotherapy [53]. (3) Reminder technology, which aimed to enhance therapy adherence without constant parental prompting, such as timer watches [54-56] and self-management reminder tools [57-59]. (4) Educational media for delivering health-related information to patients, which was subdivided into videos and other types of educational media. Videos offered visually engaging, 24/7-accessible, and anonymous dissemination of health information on topics ranging from behavioral therapy, such as bladder training and NE therapy [60-63], to preparing for surgical interventions [64]. A variety of other materials were found that provided child-friendly and engaging patient education, such as UI homework books [65,66], an NE multimedia program [67,68], and educational materials for UTIs [69-71]. (5) Telehealth and RPM for remote health information exchange, which was subdivided into communication and technological advances. Remote communication between patients and health care professionals through videoconferencing [72], home monitoring systems [73], and an embodied conversational agent [74-76] can lead to time, cost, and travel savings. Technological advances such as machine learning algorithms [79], new technologies [78,80,81], and new applications enabling home use [77,78,80-84] aimed to overcome the limitations of in-clinic procedures. (6) Enuresis alarm innovations, which were subdivided into novel configurations of traditional enuresis alarms and prevoid alarms. Enuresis alarm configurations encompassed pad-and-bell and body-worn alarms [86-88], wired and wireless systems [86], smart textiles [85,89], and a variety of alarm signals [86,90-92]. Prevoid alarms aimed to predict enuretic incidents and provide warnings in advance [93-105], with most using bladder ultrasound technology [93-104].

Many of the tools identified in this review were described as promising alternatives to traditional methods, with studies reporting their feasibility [40-46,57-59,65,66,93,95-98, 102,104,105], potential to improve outcomes [43,47-53,60, 61,64,74-76,82-84,90], and ability to save time for health care providers [49,60,61,64,68-70]. In addition, these tools were described as engaging for children, improving patient compliance [43,46,52,54-56,66,67,74-76] and leading to high levels of patient satisfaction and preference [40-46,53,58,72-77, 89,100,101,103].

However, the current body of evidence has methodological limitations, including retrospective designs [49,55,82,83]; absence of control groups [47,57,75,76,84,94,101,103]; and small sample sizes, with almost one-quarter of the studies (16/66, 24%) having a sample size of ≤15 participants [45,58,59,73,75,83,85,91,96,98-101,103-105]. While 21% (14/66) of the included studies were RCTs [43,50-52,56,60, 61,64,68,77,88-90,92], some of these (3/14, 21%) did not compare equivalent therapies [43,50,51]. Furthermore, the findings varied, with some studies (10/66, 15%) demonstrating differences in outcomes between the novel tools and traditional methods [49,55,56,74-76,88-90,94], whereas others (9/66, 14%) reported similar outcomes [40,50,60,68-70,77,87,92]. These observations highlight the need for more rigorous research to better establish the effectiveness of these tools.

### Understanding the Challenges of Managing Pediatric UI

The variability in the reported findings and gaps in robust evidence may also reflect the complexity of managing pediatric UI. This review highlights the multifaceted and multifactorial influences on pediatric UI management.

#### Multifaceted Influences

**Figure 3:**
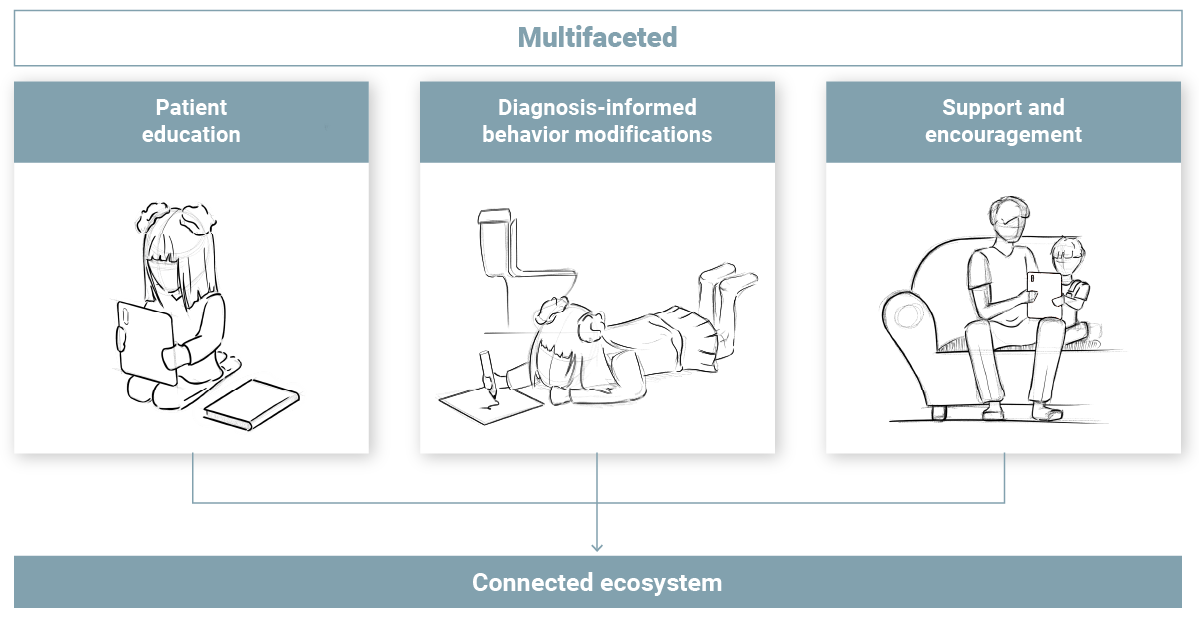
Pediatric urinary incontinence management is multifaceted, including patient education, diagnosis-informed behavior modifications, and support and encouragement. Integrating these aspects into a connected ecosystem might be an ideal way to address this multifaceted nature.

Pediatric UI management encompasses various aspects, such as patient education, diagnosis-informed behavior modifications, and providing support and encouragement (Figure 3).

Regarding patient education, the studies included in this review highlighted various educational activities, such as raising awareness [69-71], explaining complex concepts [64], setting expectations [67,68], providing lifestyle advice [60-63,75,76], and promoting adherence [53,65,66,74].

Regarding diagnosis-informed behavior modifications, management typically began with a thorough diagnostic process, as described in several included studies (9/66, 14%) [40,65,72,74-76,78,80,81]. By accurately identifying contributing factors and underlying causes, appropriate therapeutic interventions can be selected. The interventions described in the reviewed studies included providing instructions and reminders to establish healthy bladder and bowel habits [54-59]. Monitoring these habits through the collection of data on fluid intake, voiding patterns, and bowel movements [40,41,53], as well as quality of life indicators [42-45], was reported to help identify patterns and track progress over time [42-46,53,73].

Regarding support and encouragement, the included studies emphasized that support, encouragement, and feedback can help reinforce positive behaviors and maintain therapy consistency whether in the clinic [47-52], at home [42-45,72,74-77,84], or in a group setting [82].

Despite this multifaceted nature, many tools discussed in this review offered stand-alone solutions that addressed specific aspects. Examples include mobile health apps for keeping electronic bladder diaries [40,41], biogames for pelvic floor biofeedback [47-52], timer watches for timed voiding [54-56], educational videos covering specific concepts [60-64], and enuresis alarm innovations for alarm training [85-104]. While these stand-alone solutions addressed particular aspects of pediatric UI management, they did so in isolation, potentially missing opportunities to create synergies and links between different aspects of UI management. This review leads us to wonder whether integrating these various aspects into a connected ecosystem could better address the multifaceted nature of UI management.

#### Multifactorial Influences

**Figure 4:**
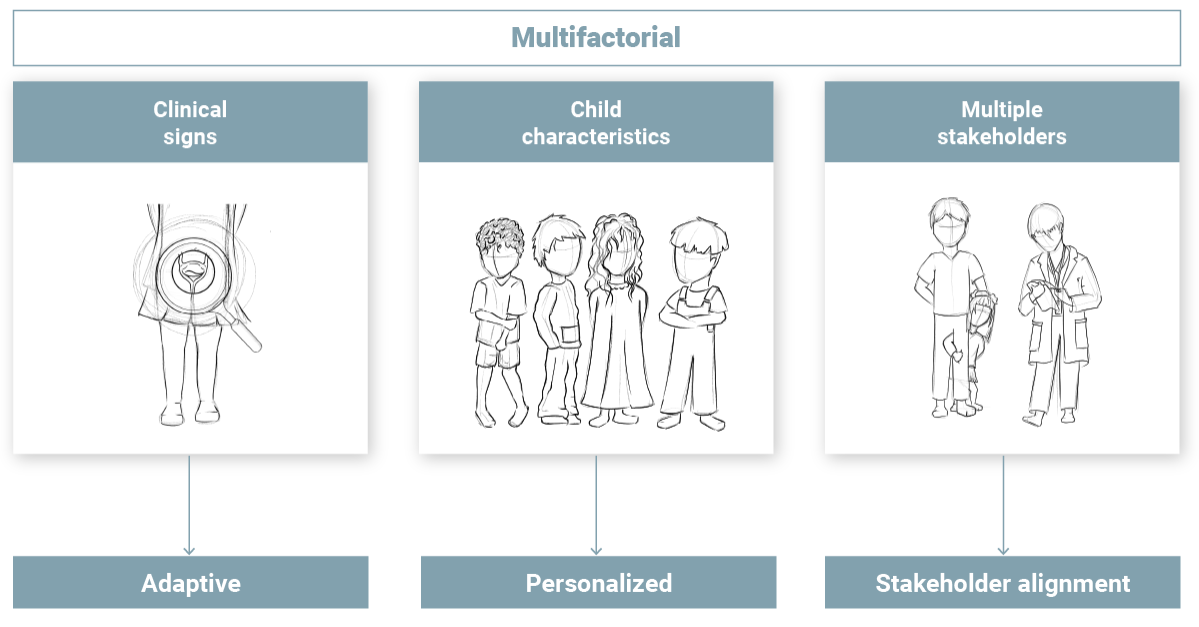
Pediatric urinary incontinence management is multifactorial, influenced by a range of factors such as (1) clinical signs that progress over time, requiring adaptive interventions; (2) child characteristics that demand a personalized approach; and (3) the involvement of multiple stakeholders whose needs must be aligned.

Pediatric UI management is influenced by a range of factors, such as clinical signs, child characteristics, and the involvement of multiple stakeholders (Figure 4).

Regarding clinical signs, the management of pediatric UI depends on the specific type and severity of symptoms that children experience. These can include multiple symptoms related to the bladder and bowel [40,60,61,72,78,80,81] occurring during the day or night [41,74-76,79]. Therefore, each child’s therapy journey is unique, with varying underlying causes, progress rates, and potential setbacks. Accurate differentiation during the diagnostic phase, along with continuous monitoring of therapy progress, as described in some studies (11/66, 17%) [42-45,53,74-77,82,83], can support tailoring management plans to the child’s specific condition and adapting them as needed. We propose that tools be adaptive to meet the evolving needs of children during therapy and help maintain their motivation. For instance, feedback from children using the urotherapy serious game indicated a desire for more dynamic gameplay that adapts to their training progress [53].

Regarding child characteristics, the management of UI depends not only on the child’s clinical signs and physiological needs but also on their psychological and emotional needs. As described in the studies, these needs can influence the child’s motivation [52,53,93,94,106], attitude toward training [43,46,55,56,65,66,74,90], and level of maturation and attention span [52,67]. Emotional factors such as self-image, embarrassment, fear, and stress were also described as key influences [59,64,87,91]. These child-specific characteristics can influence outcomes. For instance, varying findings of comparative studies on different enuresis alarm configurations [87,88] suggested that certain configurations may be more effective for some children than for others. In addition, one study reported that reminder technology was an equally effective but simpler alternative to enuresis alarms in treating DUI [54]. We propose that personalizing or selecting tools based on the child’s preferences may enhance acceptance and satisfaction and potentially lead to better outcomes.

Regarding multiple stakeholders, each stakeholder involved in pediatric UI management has distinct needs. The studies indicated that parental motivation often depended on constraints such as time, cost, and travel, prioritizing convenience and self-efficacy [22,25,40,41,46,72-76,78,89,91,93,94]. Health care professionals, on the other hand, were described as managing time constraints in busy clinical practices, aiming for efficiency and high-quality data [40,46,49,60,61,64,78,80] while also maintaining empathy and establishing a therapeutic alliance [72,75]. Aligning these diverse needs can be challenging, as described for the UTI remote monitoring system [73]. Although the system comprised a set of products, most parents primarily used the urinalysis device because it met their needs for self-efficacy and reassurance. The other products were underused, leading to frustration among health care providers, who faced added workload for setting up the system without improved data quality. These findings underscore the importance of designing tools that balance the needs of all stakeholders, improving quality of life for patients and families while optimizing time and efficiency for health care professionals.

### Transforming Health Care With Connected Ecosystems

Given the multifaceted and multifactorial nature of pediatric UI management, we propose that novel tools should be designed as health care ecosystems that are (1) connected, (2) adaptive, (3) personalized, and (4) aligned with the needs of all stakeholders.

The concept of health care ecosystems for managing pediatric UI is rooted in a broader transformation of the health care industry driven by innovation [115]. Advances such as electronic health records, networked computer systems, and telehealth have revolutionized health care delivery, shifting from a model centered on face-to-face interactions with individual clinicians to a smart, interconnected health care community emphasizing shared decision-making [116]. The COVID-19 pandemic further accelerated this transition, highlighting the critical role of digital technologies in maintaining access to health care [117]. Furthermore, recent breakthroughs in computational power and artificial intelligence promise to expand the capabilities of health care systems even further [116].

A fair part of the currently available medical devices already has the ability to connect and communicate with other devices or systems. This connectivity transforms previously isolated tools into collaborative systems that operate within closed feedback loops, tailoring outputs to the individual [118]. For example, in glucose monitoring, sensors measure blood glucose levels, which then inform insulin delivery systems to maintain optimal glucose control [119]. Similarly, smart pillboxes improve medication adherence by tracking remaining doses, providing reminders through a connected alarm device [120].

While many of these systems have been tested primarily in contexts such as monitoring older patients at home [121]—where they support independence and reduce health care burdens—pediatric urology presents a significant opportunity for similar innovations. For example, a recently published article on the Minze Homeflow system demonstrates how a connected ecosystem can enhance pediatric UI management [122]. It integrates physical products (uroflowmeter and accessories) with digital tools (Minze app and clinician portal) for remote monitoring of uroflowmetry and bladder diary data. By shifting diagnostics to the home environment, the Homeflow system minimizes stress for pediatric patients while providing more reliable data compared to in-clinic measurements. Such a system demonstrates the feasibility and benefits of connected health care ecosystems in addressing specific challenges in pediatric urology.

Another study demonstrated how the Homeflow ecosystem could be further expanded by connecting additional products to the system, such as a smart drinking bottle and reminder technology, to personalize urotherapy [123]. The smart drinking bottle and home uroflowmeter monitored fluid intake and urinary output, whereas a reminder watch linked these devices, offering personalized voiding prompts based on the child’s bladder capacity and fluid intake. This demonstrates the potential of connected ecosystems to adapt and align with the individual care needs of pediatric patients.

### Overcoming Challenges in Health Care Innovation

The successful implementation of health care innovation and ecosystems requires addressing important challenges, such as regulatory and privacy concerns, equitable access, screen time considerations, and keeping health care human centered. Thoughtful strategies are needed to overcome these challenges and maximize benefits.

#### Regulatory and Privacy Concerns

Ensuring patient privacy and data security remains a critical challenge for digital health care innovation [124]. As these tools handle sensitive patient data, robust regulatory frameworks must be established to protect patient data. However, balancing innovation with regulation remains a challenge, especially considering the rapid pace of technological advancements. Current regulations may struggle to keep up, potentially delaying the adoption of transformative tools. A balanced approach is required to ensure safety and trust without hampering innovation that can improve patient care.

#### Equity in Access

Ensuring equitable access is another challenge for health care innovation, especially in low-resource settings. Individuals in such settings must not be left behind as they are often the ones who stand to benefit the most from these advancements [115]. However, barriers such as limited infrastructure, potentially high entry costs, and digital literacy disparities can hinder adoption [116]. Nevertheless, innovation also has the potential to improve equity by decentralizing care. Shifting diagnostics and therapies from clinics to homes or communities can reduce costs and improve accessibility. In addition, customizable solutions tailored to varying resource levels, aligned with reimbursement models, or supported through public health initiatives can support the equitable implementation of innovation.

#### Screen Time Considerations

The use of digital tools raises concerns about screen time and its potential impact on children’s health. A 2019 systematic review [125] identified associations between excessive screen time and health risks, including adiposity, unhealthy diet, depressive symptoms, and reduced quality of life. However, the review did not consider the influence of content or context on these outcomes. Research indicates that well-designed, age-appropriate content, especially when curated by engaged adults [126], can positively impact learning and behavior [127]. For example, screen time can calm distressed children during medical procedures [128] or support language learning [129]. Moreover, active video games or nature exploration apps can encourage physical activity and imaginative play by connecting on- and off-screen experiences [130].

Nevertheless, certain screen use patterns present clear risks. Screen time before bedtime is strongly associated with sleep disturbances [131]. The mere presence of electronic devices in bedrooms has been linked to shorter sleep duration, partly due to melatonin suppression [132]—an essential consideration for tools addressing NE. A 2024 systematic review [133] reinforced these findings, showing that greater use of mobile devices, particularly at bedtime or in patterns of device dependence, can harm mental health in children and adolescents. At the same time, it emphasized technology’s dual impact—while social media often correlates with negative effects, other communication tools can enhance psychological well-being by strengthening social connections and providing access to support. These effects vary across individuals, highlighting the need to identify those most vulnerable to adverse effects.

In summary, the impact of screen time is shaped by multiple factors. To maximize benefits, digital tools should be thoughtfully designed to deliver engaging, age-appropriate content; promote balanced screen habits; integrate on- and off-screen interactions; and avoid bedtime use and overreliance on devices.

#### Keeping Health Care Human Centered

Digital technology presents significant opportunities to enhance efficiency and accuracy in health care. However, human interaction remains fundamental to preserving empathy, trust, and nuanced clinical decision-making. For example, a study in this review reported that health care providers experienced difficulties building rapport with pediatric patients due to technical barriers [72]. This underscores the importance of designing tools that augment health care professionals’ capabilities, ensuring that technology supports rather than depersonalizes patient care. Therefore, a human-centered approach to technology is needed to actively incorporate the perspectives and needs of patients, caregivers, and health care providers, placing them at the center of smart and connected health care in both research and practice [116].

Such an approach values not only objective evidence but also subjective patient and stakeholder experiences, combining quantitative and qualitative methods. However, in our review, only 12% (8/66) of the studies used qualitative [42,72,99] or mixed methods [40,44,58,67,73] designs. Despite this, more than one-third (25/66, 38%) of studies evaluated outcomes related to patient experience, such as satisfaction, motivation, and usability [42-45,52,53,57,58,66,67,71-73,75,76,82,88-90, 94,98-101,103]. This indicates a growing recognition of the importance of patient-centered outcomes but also underscores the need for more qualitative approaches to capture deeper insights.

To incorporate a human-centered approach, we conducted a separate qualitative study alongside this scoping review to explore children’s perspectives, experiences, and expectations regarding urotherapy-supporting tools. To facilitate this, we developed a focus group discussion toolkit specifically tailored to pediatric UI [134]. The toolkit incorporated creative tasks involving imagination and play, enabling children to express their thoughts and experiences in a child-friendly and engaging manner. By combining insights from this scoping review with the findings of the focus groups, we aimed to provide a comprehensive perspective that blends existing evidence with the desires and needs of the target population. This integrated approach can guide the development of more effective and user-centered tools for pediatric UI management.

### Limitations

This scoping review has certain methodological limitations that should be considered when interpreting its results. First, the search was limited to 3 electronic databases, and only English-language papers were included. Second, only 1 reviewer screened the entire set of search results. However, the team engaged in frequent and extensive discussions throughout the study selection process to ensure rigorous screening. Third, our search had an extensive scope due to our broad criteria for defining innovative, technology-driven, digital tools, as well as having no time limits. The broad field and the potential abundance of relevant tools made it challenging to provide a concise overview of all the information. Consequently, this review encompassed a broad range of studies and a wide variety of innovative, technology-driven, digital tools.

### Conclusions

This scoping review offers valuable insights into various categories of innovative, technology-driven, digital tools for managing pediatric UI and their supporting evidence. The inclusion of 66 articles highlights a considerable level of innovation in the field.

We identified 6 main categories of tools: digital self-management, serious games, reminder technology, educational media, telehealth and RPM, and enuresis alarm innovations. Further subcategories were found within these main categories, such as video and other media within educational media, communication and technological advances within telehealth and RPM, and novel configurations and prevoid alarms within enuresis alarm innovations.

Many of these tools were described as promising alternatives to traditional methods, with studies reporting feasibility, effectiveness in improving outcomes, and potential time savings for health care providers. In addition, these tools were described as engaging children effectively, potentially enhancing patient compliance and contributing to high patient satisfaction and preference. However, this review identified gaps in research, highlighting the need for more rigorous research to better assess the effectiveness of these tools and address the complex, multifaceted challenges of pediatric UI management. We propose the development of connected, adaptive, and personalized health care ecosystems that integrate diverse strategies for UI management, including education, diagnosis, therapy, monitoring, and feedback. By enabling synergies and links between these different strategies, such ecosystems could create a closed-loop system that enhances the alignment and efficiency of care.

We suggest that future research and development in this field adopt a human-centered and multidisciplinary approach, combining quantitative and qualitative methods to incorporate user perspectives and involve all stakeholders from the start. This mixed methods approach may help ensure that both objective evidence and subjective experiences are equally valued, supporting impactful innovations in the complex field of pediatric UI management.

## Appendix

Multimedia Appendix 1 PRISMA-ScR checklist.

Multimedia Appendix 2 Full search strategy.

Multimedia Appendix 3 Overview of the included studies by tool category.

## Abbreviations

BBD: bladder and bowel dysfunction
DUI: daytime urinary incontinence
DV: dysfunctional voiding
EMG: electromyography
NE: nocturnal enuresis
OAB: overactive bladder
PRISMA-ScR: Preferred Reporting Items for Systematic Reviews and Meta-Analyses extension for Scoping Reviews
RCT: randomized controlled trial
RPM: remote patient monitoring
UI: urinary incontinence
UTI: urinary tract infection

## Data Availability

The datasets generated or analyzed during this study are available from the corresponding author on reasonable request.
