# Supplementary material for: Innovative, Technology-Driven, Digital Tools for Managing Pediatric Urinary Incontinence: Scoping Review
Source: Interact J Med Res. 2025 May 5;14:e66336. doi: 10.2196/66336 (PMC12089871; doi:10.2196/66336)
Supplement: Multimedia Appendix 2 [file ijmr_v14i1e66336_app2.docx]

# Full search strategy

List of keywords combined by the operator "OR" for each search concept: (ia) population age group, (ib) population dysfunction, (ii) concept, and (iii) context. These search concepts are subsequently combined using the operator "AND" for the overall search.

|  | Population | | Concept | Context |
| --- | --- | --- | --- | --- |
|  |  | |  |  |
| Search String | Child OR Children OR Adolesc* OR Pediatric OR Paediatric OR Teenager | Lower urinary tract symptoms OR ((Bladder OR Bowel OR Bladder bowel) AND dysfunction) OR ((daytime OR day OR nighttime) AND (incontinence OR wetting)) OR ((Diurnal OR Nocturnal) AND Enuresis) OR Bedwetting OR ((Overactive OR Underactive) AND bladder) OR Voiding postponement OR Dysfunctional voiding OR Urinary Tract Infection OR Spina Bifida | Smart OR Intelligent OR Digital OR Auto* OR Device OR Tool OR Technol* OR mHealth OR eHealth OR App OR Smartphone OR Mobile phone OR Tablet OR Game OR Gamification OR Video OR Multimedia OR Bluetooth OR Internet OR Tele* OR Remote monitoring OR Interact* OR Innovat* OR Personali* OR Animat* OR Mobile | Urotherapy OR Bladder Training OR Educati* OR Informati* OR Communicat* OR Biofeedback OR (Alarm AND (therapy OR training OR treatment)) OR ((Timed OR scheduled) AND (voiding OR drinking)) OR Self-management OR Self-monitoring OR ((Voiding OR Bladder OR Urinary OR Bowel OR incontinence) AND diary) OR ((Voiding OR Frequency Volume) AND chart) OR behavior* management |
| Search Fields | Title/Abstract | All Fields | All Fields | All Fields |
