# Supplementary material for: Innovative, Technology-Driven, Digital Tools for Managing Pediatric Urinary Incontinence: Scoping Review
Source: Interact J Med Res. 2025 May 5;14:e66336. doi: 10.2196/66336 (PMC12089871; doi:10.2196/66336)
Supplement: Multimedia Appendix 3 [file ijmr_v14i1e66336_app3.docx]

#
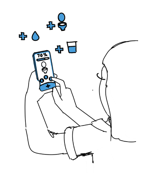
Overview of the included studies by tool category

#### 1. Digital self-management

Table S1. Studies categorized under mobile health (n=7)

| **Authors** | **Study design** | **Population** | **Tool** |
| --- | --- | --- | --- |
| Johnson et al. (2014) | Mixed Methods | BBD,  4-17y,  N=83 | This web app, accessible on any mobile device with an internet browser, allows patients to record their urinary and bowel habits in real time. Entries are automatically uploaded and securely stored on a research server, with the option to download the data as a PDF file. |
| Myint et al. (2016) | Review | / | Smartphone applications available on iTunes, Android Play Store, and BlackBerry World that include a bladder diary function and are related to the investigation, follow-up, and treatment of childhood enuresis. |
| Whale et al. (2021) | Qualitative | DUI,  10-19y,  N=23 | UrApp is a smartphone app that helps adolescents with UI manage their fluid intake by setting daily drinking goals and sending reminders. It tracks and records fluid intake, toilet visits, medication, and mood, while showing progress through charts. The app also offers rewards for meeting goals and provides personalized feedback to encourage positive habits. |
| Bonnert et al. (2017) | RCT | Bowel dysfunction,  13-17y,  N=101 | This internet-delivered cognitive behavior therapy program for adolescents with irritable bowel syndrome includes 10 weekly modules with short texts, audio files, and videos, each ending in exposure-based homework exercises. There are 5 additional modules for parents to help them encourage their child. |
| Choi et al. (2019) | Quasi-experiment | Neurogenic,  7-12y,  N=5 | The "Glowing Stars" mHealth program helps children with spina bifida self-manage their health by monitoring voiding, defecation, skin care, medication, and mood. It uses gamification with star-charts and progress graphs to encourage daily self-monitoring. |
| Choi et al. (2020) | Mixed Methods | Neurogenic,  7-12y,  N=36 | Identical to the above. |
| Fine et al. (2009) | Quasi-experiment | UTI,  >12y,  N=232 | ParentLink is a parent-driven tool for pediatric emergency medicine, focusing on conditions like UTIs. It allows parents to enter data on their child’s symptoms, medications, and allergies, ensuring that critical information is not overlooked. The tool provides tailored, evidence-based recommendations to support clinical decision-making. |


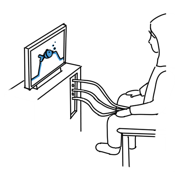


#### 2. Serious games

Table S2. Studies categorized under serious games (n=7)

| **Authors** | **Study design** | **Population** | **Tool** |
| --- | --- | --- | --- |
| McKenna et al. (1999) | Prospective n.c | DV,  5-11y,  N=41 | Several biogames were used, including a golf game, spaceship game, elephant ride, and basketball game, where the game action is controlled by the patient's pelvic floor muscle activity through contractions and relaxations. |
| Herndon et al. (2001) | Quasi-experiment | DV,  4-13y,  N=53 | An interactive, computer game-assisted pelvic floor retraining (biogame) was used. |
| Kaye et al. (2008) | Retrospective study | DV,  Mean 7y,  N=120 | A biogame was used in which the patient controls a dolphin’s movements, such as jumping through hoops, by using their pelvic floor muscles. |
| Kajbafzadeh et al. (2011) | RCT | DV,  5-16y,  N=80 | Several biogames were used, including Dolphin, Monkey, Fish, Bee, and UFO, where the characters' movements reflect the child's pelvic floor muscle activity. |
| Ladi-Seyedian et al. (2015) | RCT | UAB,  5-16y,  N=50 | Identical to the above, but for UAB. |
| Oktar et al. (2018) | RCT | DV,  4-15y,  N=40 | Several biogames were used in the interactive biofeedback sessions, with default animations including sea, forest, and diagram graphs. |
| Nieuwhof-Leppink et al. (2019) | Quasi-experiment | DUI,  8-14y,  N=50 | In the serious game, children embark on a mission to save Princess Mina from monsters. Lives are earned by completing a daily bladder diary, with bonus hearts for staying dry or voiding more than 7 times a day. These rewards enable them to purchase fighting equipment to battle the monsters. The game includes training videos on bladder function and tracks progress over 90 days. Each day is represented by a bullet on the landscape, with new monsters and changing scenery after each week of training. |


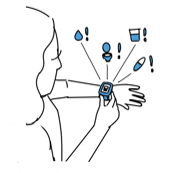


#### 3. Reminder technology

Table S3. Studies categorized under reminder technology (n=6)

| **Authors** | **Study design** | **Population** | **Tool** |
| --- | --- | --- | --- |
| Hagstroem et al. (2008) | Retrospective study | DUI,  Mean 7.8y,  N=240 | A programmable timer watch was used for timed voiding, sounding an alarm according to the programmed times. |
| Hagstroem et al. (2010) | RCT | OAB,  5-14y,  N=58 | A timer watch, Triax 35 by Nike Inc., was used, featuring seven programmable alarms to assist with timed voiding. |
| Halliday et al. (1987) | Quasi-experiment | DUI,  5-15y,  N=44 | One system was a sham wetting alarm, essentially a timer watch, while the other triggered an alarm when wetting occurred. Both were visually identical, using a flat plastic sensor attached to underpants and connected via wires to a small alarm box, worn on a belt or in a pocket. |
| Flannery et al. (1997) | Case study | Neurogenic,  17y,  N=1 | EasyAlarms is a laptop calendar program that helps individuals with prospective memory disability remember daily tasks like self-catheterization and medication. The program flashes the screen and sounds alarms to prompt users, who then log their responses, which are available for review by users, caregivers, and professionals. |
| Jennings et al. (2020) | Mixed Methods | Neurogenic,  3-7y,  N=4 | The HeyJoy Octopus watch is a child-friendly smartwatch that supports daily routines for children with Spina Bifida. It uses visual icons for self-care, play, chores, and mealtimes, and includes a built-in fitness tracker to promote activity. The watch syncs with a smartphone app and includes a reward system where children earn stars for completing tasks and can gain extra rewards by beating a timer. |
| Bartos et al. (2021) | Prospective n.c | (Neuro)-  developmental disorder,  4-10y,  N=21 | Potty Monkey is an interactive toy designed for time-based toilet training, modeling normal toileting behavior for children with special needs. It prompts children to place the Monkey on its toilet at set intervals, responds with delight when used correctly, and indicates an accident if ignored after three warnings. |

####
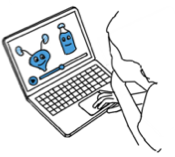
4. Educational media

##### 4.1 Video

Table S4. Studies categorized under the subcategory of video in educational media (n=5)

| **Authors** | **Study design** | **Dysfunction** | **Tool** |
| --- | --- | --- | --- |
| Braga et al. (2017) | RCT | BBD,  5-10y,  N=150 | The 7-minute animated bladder training video features “Becky the Bladder” and “Will the Water Bottle.” It explains urinary tract anatomy and function, covers symptoms like incontinence, UTIs, and constipation, and emphasizes three key bladder training strategies: timed and double voiding, adequate water intake, and managing constipation. |
| Brownrigg et al. (2017) | RCT | BBD,  5-10y,  N=150 | Identical to the above. |
| Batuman et al. (2016) | RCT | Anatomical,  5-12y,  N=42 | The preoperative video for (genito-urinary) surgery features an 11-year-old girl, her mother, and hospital staff, using role-play modeling to guide children through the perioperative process. It shows each step from admission to recovery, including a demonstration of the face mask anesthesia procedure on a teddy bear, with ongoing reassurance and support from the care team. |
| Toprak et al. (2021) | Review | / | YouTube videos providing medical information to patients using the keyword "Nocturnal Enuresis" were analyzed. |
| Nishizaki et al. (2021) | Review | / | YouTube videos providing medical information to patients using the keyword “Ya-nyou-shou,” the Japanese term for nocturnal enuresis, were analyzed. |


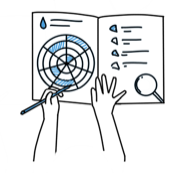


##### 4.2 Other media

Table S5. Studies categorized under the subcategory of other in educational media (n=7)

| **Authors** | **Study design** | **Population** | **Tool** |
| --- | --- | --- | --- |
| Bower et al. (2001) | Validation | OAB,  5-12y,  N=86 | This tool features child-focused measures for assessing urgency and incontinence severity, engaging children as "bladder detectives." It includes two 10-unit visual analog scales for urgency and a "Dry Pie" homework book, where children color a pie chart with color-codes to log the severity of daily incontinence. |
| Rhodes et al. (2008) | Quasi-experiment | DUI,  6-10y,  N=20 | This home workbook guides children through a step-by-step bladder rehabilitation program to help them achieve dryness.  The two-week plan includes information for parents, lessons for the child, and exercises like learning to "hold on" to urine and increasing fluid intake. |
| Evans et al. (1998) | Mixed methods | NE,  8-10y,  N=43 | "All About Nocturnal Enuresis" is a multimedia touch-screen program for bedwetting education, featuring simple text, drawings, cartoons, and voiceovers. It includes seven 10-minute modules on bladder function, causes of bedwetting, self-help strategies, potential treatments, and parental advice. Progress through the modules is tracked with a passport as children work toward becoming the "boss of their bladder." |
| Redsell et al. (2003) | RCT | NE,  5-16y,  N=270 | Identical to the above. |
| Teo et al. (2016) | Quasi-experiment | UTI,  <2y,  N=621 (urine samples) | This UTI educational tool comprised a pre-made urine collection kit, which included a urine specimen jar, cleaning wipes, an underpad, and a handout detailing the clean-catch urine collection method. |
| Jacob et al. (2020) | Quasi-experiment | UTI,  median 3y,  N=2104 (urine samples) | This UTI educational tool was similar to the one mentioned above. Awareness was reinforced by posters in all areas where urine is ordered or collected, with kits placed near these posters in stocked bins. |
| Kaur et al. (2020) | Validation | UTI,  12-15y,  N=25 | The Foldscope is a low-cost, origami-based microscope designed for educational purposes, such as raising awareness about genital hygiene and UTIs in schools. Assembled from a flat sheet of paper in under 10 minutes, it holds a specimen slide and allows a smartphone to be attached for detailed observation and UTI detection with submicron resolution. |

#### 5. Telehealth and RPM
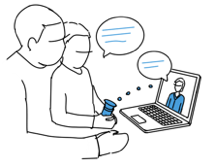


##### 5.1. Communication

Table S6. Studies categorized under the subcategory of communication in telehealth

and RPM (n=5)

| **Authors** | **Study design** | **Population** | **Tool** |
| --- | --- | --- | --- |
| Kuo et al. (2021) | Qualitative  (online survey) | / | TeleBBD refers to video conferencing used for managing bladder and bowel dysfunction through remote patient visits. |
| Carter et al. (2019) | Mixed methods | Neurogenic,  <5y,  N=10 | This telehealth intervention allows parents of children with neurogenic bladder to monitor UTIs at home using a portable urinalysis device, Bluetooth weight scale, thermometer, and an electronic voiding/bowel diary. Data is transmitted via a mobile device to the clinical team, who provide alerts and messages about potential UTIs. |
| Baker et al. (2014) | Design-based research | / | eADVICE and Dr. Evie are complementary digital tools for managing pediatric UI while waiting for specialist visits. eADVICE provides personalized treatment and lifestyle advice based on medical history and a bladder diary, sending reports to the GP for support. Dr. Evie, an embodied conversational agent (culturally neutral, empathetic female character), engages patients through interactive conversations and goal-setting to build trust and encourage adherence to treatment plans. |
| Richards et al. (2016) | Prospective n.c | Daytime and/or nighttime UI,  5-18y, N=14 | Identical to the above. |
| Richards et al. (2018) | Prospective n.c | Daytime and/or nighttime UI,  6-16y, N=74 | Identical to the above. |

#####
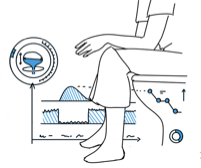
5.2 Technological advances

Table S7. Studies categorized under the subcategory of technological advances in

telehealth and RPM (n=8)

| **Authors** | **Study design** | **Population** | **Tool** |
| --- | --- | --- | --- |
| Tokar et al. (2021) | Modelling study | Daytime and/or nighttime UI,  5-12y,  N=8071 | A machine learning algorithm for predicting enuresis in children uses a logistic regression model with 14 features, reduced from 34 variables. Key factors include age at toilet training onset, urinary urgency, holding maneuvers, defecation frequency and family history of enuresis. |
| Urbona-vicius et al. (2016) | Review | / | Several methods are used to measure urinary flow rate: gravimetric methods weigh collected urine, volumetric techniques use fluid filling and draining speeds in specialized vessels, mechanical methods detect forces from urine impact (e.g. spinning disk or float-based devices), electrical methods detect changes in electrical properties (e.g. capacitance or magnetic fields), image processing methods analyze flow patterns using optical sensors or X-rays, and ultrasonic, vibration, and acoustic methods use sound waves and vibrations (e.g. Doppler effect or sonouroflowmetry). |
| Lee et al. (2021) | Validation | BBD,  ≤18y,  N=16 | The PRIVY Urine Flow Monitor app (Soundable Health) uses a smartphone to record the sound of urine hitting the toilet bowl or water. It processes these audio signals with machine learning algorithms, including background noise removal and urination sound classification, to generate a uroflow curve. |
| Macnab et al. (2012) | Review | / | NIRS is a non-invasive optical technology that employs near-infrared light to monitor blood flow and tissue oxygenation in real time, offering insights into physiological changes such as muscle contraction and organ function. Positioned on the abdominal skin over the bladder, it employs either laser systems with fiber-optic cables or compact, wireless LED systems with built-in memory and Bluetooth for data transfer. |
| Schulman et al. (2001) | Retrospective study | DV,  4-15y,  N=102 | Two biofeedback training methods were used: uroflowmetry biofeedback in a group setting, where 3-4 children void 4 to 8 times in 6 hours on a uroflowmeter with real-time coaching from a nurse; and individual EMG biofeedback, where children perform pelvic floor exercises in front of a monitor displaying EMG readings for 45 to 90 minutes. |
| Klijn et al. (2006) | RCT | DV,  6-16y,  N=143 | A battery-operated, portable uroflowmeter connected to a computer was used for home biofeedback, offering real-time display and storage of urinary flow data. It is paired with a foldable plywood toilet chair adjustable to 30, 35, and 40 cm heights, designed to fit most children without the need for a footrest. |
| Sugar et al. (1982) | Retrospective study | DV,  6-16y,  N=10 | Urodynamic biofeedback combines real-time urinary flow measurements with pelvic floor EMG feedback. A promising new home EMG biofeedback device was introduced, still under investigation: Battery-operated and discreetly worn under clothing, it features a watch-like design with a row of lights that indicate pelvic floor muscle activity on a scale from low to high. |
| Ferroni et al. (2017) | Prospective n.c. | NE,  5-18y,  N=22 | A TENS device (LGMedSupply) was used for home treatment after a 5-minute in-office test for tolerability. Children received 60 minutes of foot stimulation each evening. This cost-effective device and its replacement pads are commercially available, making it easily accessible. |

6. Enuresis alarm innovations
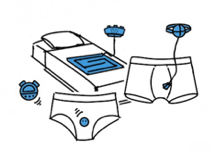


##### 6.1 Novel configurations

Table S8. Studies categorized under the subcategory of novel configurations in enuresis

alarm innovations (n=8)

| **Authors** | **Study design** | **Population** | **Tool** |
| --- | --- | --- | --- |
| Parkova et al. (2013) | Review | / | Enuresis alarms are available in various configurations: pad-and-bell alarms feature a bed mat with conductive material, body-worn alarms have sensors close to the body and can be flexible or rigid, wired or wireless, and smart textile sensors integrate conductive threads into underpants. Alarm signals can be acoustic, light-based, vibrating, or use personalized messages. |
| Fordham et al. (1989) | Quasi-experiment | NE,  6-16y,  N=56 | Two enuresis alarms were used: the SM1 pad and bell alarm, which has a large detector sheet linked to a box that sounds an alarm and flashes a red light; and the Mini Dri-Nite body-worn alarm, which uses a tiny sensor, placed in a pad worn in the underpants, triggering a high-pitched sound through a small unit attached to the child's clothes. |
| Peck et al. (2020) | RCT | NE,  6-16y,  N=86 | Two enuresis alarms were used: the P Model bell-and-pad alarm by Ramsey-Coote Instruments and the WetAlert body-worn alarm by Ferring Pharmaceutics. |
| Manson (1979) | Validation | (Neuro)-  developmental disorder,  N=4 | This low-cost portable enuresis alarm (90 x 60 x 30 mm; 70g) features a disposable pad with conductive rubber-coated terylene strips and absorbent soft paper for comfort. Designed for safety, it maintains a low current to prevent skin irritation and provides reliable resistance detection to minimize false and missed alarms. |
| Mruzek et al. (2019) | RCT | (Neuro)-  developmental disorder,  3-6y,  N=32 | This wireless moisture pager includes a disposable urine sensor pad with silver-coated threads, worn in the child's undergarment. It connects to a battery-powered, water-resistant transmitter via stainless steel snaps, which syncs via Bluetooth with the Quicktrainer iOS app for time-stamped alerts and toilet training reinforcement support. |
| Caldwell et al. (2016) | RCT | NE,  6-18y,  N=353 | The code-word enuresis alarm features a wireless moisture sensor and an alarm box with a digital voice recorder for parents to record daily personalized code words. Upon waking and deactivating the alarm triggered by wetness, the child hears the pre-recorded code word and is encouraged to use the toilet. The child is rewarded for recalling the code word the next morning. |
| Elinder et al. (1985) | RCT | NE,  ≥7y,  N=53 | Uristop is a device with a thermostatic sensor placed near the urinary meatus, activated by the warmth of the first drops of urine. It sends an electric impulse to two electrodes placed suprapubically, inducing muscle contraction and stimulating the normal impulse from the sphincter. |
| Barroso et al. (2014) | Validation | NE,  7-20y,  N=6 | This device uses a humidity sensor to trigger an electric impulse through two surface electrode pads placed perineally. This activates the external sphincter to interrupt urination and may reflexively relax the bladder. If not turned off after 20 seconds, it emits a sound to wake the child. |

#####
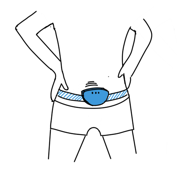
6.2. Prevoid alarms

Table S9. Studies categorized under the subcategory of prevoid alarms in enuresis

alarm innovations (n=13)

| **Authors** | **Study design** | **Population** | **Tool** |
| --- | --- | --- | --- |
| Petrican et al. (1998) | Validation | NE,  3-15y,  N=41 | This prevoid alarm uses a single ultrasound crystal to detect echoes from the posterior bladder wall as urine creates an echo-free zone. When bladder volume reaches 80% capacity, the echo amplitude triggers the alarm. A belt ensures precise probe positioning for accurate detection, while the compact 38 cm² PCB fits comfortably under the child's clothing during sleep. |
| Beauchamp-Parent et al. (1998) | Validation | Bladder phantom | This updated prevoid alarm (see above) features an improved detection method to better handle probe movement. It synchronizes the measurement cycle with the child’s respiratory rhythm and activates only when bladder depth exceeds a user-set threshold, accommodating variations in bladder and body morphology, including damping from fat. |
| Pretlow (1999) | Quasi-experiment | NE,  6-16y,  N=40 | This prevoid alarm uses a modified PCI 5000 ultrasound device worn with an elastic garment. It measures bladder volume every 15 minutes and triggers an alarm when a set threshold is reached. The bedside unit includes an acoustic alarm, optional vibration alarm, and a wireless remote alarm for parents. |
| Kristiansen et al. (2004) | Validation | NE,  N=1 | This prevoid alarm features a wearable, compact device (16x16x15 mm) with a seven-phased ultrasonic array transducer arranged in a circle for optimal bladder detection. It connects via Bluetooth to a laptop for data processing, displaying measurements and trends, and triggering alarms when the bladder volume exceeds a preset level. |
| Padmapriya et al. (2015) | Validation | Bladder phantom | This prevoid alarm integrates an Arduino microcontroller with a disc-shaped ultrasonic array transducer mounted on Velcro for a flexible, wearable probe. The Arduino calculates bladder volume via ellipsoid equations based on ultrasound data, displays it in real-time on an LCD, and triggers an alarm when the volume exceeds a user-set threshold, with notifications sent wirelessly. |
| Kuru et al. (2019) | Validation | NE,  6-9y,  N=8 | MyPAD is a prevoid alarm system that combines four ultrasound sensors with artificial intelligence to estimate bladder volume. It is integrated into a comfortable lycra-based garment with a sticky gel pad. A WiFi-connected bedside alarm box analyzes the data, triggers alerts, and provides user feedback through a simple interface with buttons for true and false alarms. This feedback, along with a moisture sensor, enables the system to self-learn and adjust. |
| Caswell, Kuru et al. (2020) | Qualitative | NE,  N=3 | Identical to the above. |
| Kuru et al. (2020) | Validation | NE,  7-14y,  N=12 | Identical to the above. |
| van Leuteren et al. (2017) | Validation | DV,  6-12y,  N=14 | URIKA is a prevoid alarm with a single-element ultrasound transducer (90 × 72 × 21 mm) and an electronic case (165 × 80 × 28 mm) for signal processing and Bluetooth data transfer, both mounted on an elastic belt around the lower abdomen. It calculates the distance between the anterior and posterior bladder walls using a software algorithm and triggers an alarm when this distance exceeds a preset threshold, signaling a full bladder. |
| van Leuteren et al. (2018) | Validation | DUI,  6-12y,  N=30 | SENS-U is a compact, redesigned version of URIKA (see above), integrating four ultrasonic transducers and electronics into a single device (95 x 55 x 16 mm). It attaches to the lower abdomen with skin-friendly adhesive and ultrasound gel. Real-time bladder status is sent via Bluetooth to a mobile app using weather symbols. When the bladder reaches 80% capacity, based on a threshold set by a medical professional, the SENS-U vibrates and the app displays a cloud icon to alert the child. |
| van Leuteren et al. (2019) | Prospective n.c | DUI,  6-16y,  N=15 | Identical to the above. |
| Kwinten, van Leuteren et al. (2020) | Prospective n.c | NE,  6-12y,  N=15 | Identical to the above. |
| Moon et al. (2017) | Modelling Study | NE,  N=1 | This prevoid alarm system predicts enuretic incidents using a deep learning algorithm and multiple physiological signals from wearable devices: a customized belt measures bladder volume via impedance (key predictor), a smartwatch tracks heart rate (critical predictor) and sleep stages (preliminary alert), and fabric bands on both dorsums of the foot detect periodic limb movements (preliminary alert). |
